# Modeling Human Morphological Competence

**DOI:** 10.3389/fpsyg.2020.513740

**Published:** 2020-11-12

**Authors:** Yohei Oseki, Alec Marantz

**Affiliations:** ^1^Faculty of Science & Engineering, Waseda University, Tokyo, Japan; ^2^Department of Linguistics, New York University, New York, NY, United States; ^3^Department of Psychology, New York University, New York, NY, United States; ^4^NYU Abu Dhabi Institute, New York University, Abu Dhabi, United Arab Emirates

**Keywords:** morphology, grammaticality, acceptability, probability, psycholinguistics, computational modeling

## Abstract

One of the central debates in the cognitive science of language has revolved around the nature of human linguistic competence. Whether syntactic competence should be characterized by abstract hierarchical structures or reduced to surface linear strings has been actively debated, but the nature of morphological competence has been insufficiently appreciated despite the parallel question in the cognitive science literature. In this paper, in order to investigate whether morphological competence should be characterized by abstract hierarchical structures, we conducted a crowdsourced acceptability judgment experiment on morphologically complex words and evaluated five computational models of morphological competence against human acceptability judgments: Character Markov Models (Character), Syllable Markov Models (Syllable), Morpheme Markov Models (Morpheme), Hidden Markov Models (HMM), and Probabilistic Context-Free Grammars (PCFG). Our psycholinguistic experimentation and computational modeling demonstrated that “morphous” computational models with morpheme units outperformed “amorphous” computational models without morpheme units and, importantly, PCFG with hierarchical structures most accurately explained human acceptability judgments on several evaluation metrics, especially for morphologically complex words with nested morphological structures. Those results strongly suggest that human morphological competence should be characterized by abstract hierarchical structures internally generated by the grammar, not reduced to surface linear strings externally attested in large corpora.

## 1. Introduction

Chomsky (1957) seminally argued that the grammar categorically generates *grammatical* sentences of the language, while speakers gradiently judge *acceptable* sentences of the language, as summarized below:

“The fundamental aim in the linguistic analysis of a language L is to separate the *grammatical* sequences which are the sentences of L from the *ungrammatical* sequences which are not sentences of L and to study the structure of the grammatical sequences. The grammar of L will thus be a device that generates all of the grammatical sequences of L and none of the ungrammatical ones.” (Chomsky, 1957, p. 13; emphasis original)

On this internalist view, syntactic competence should be characterized by abstract hierarchical structures internally generated by the grammar (Everaert et al., 2015; Ott, 2017), where grammaticality and acceptability correspond to linguistic representation and processing, respectively, hence the familiar competence-performance distinction. The independence of the grammar from probabilities over surface linear strings was evidenced by the famous *Colorless green ideas sleep furiously* sentence, which is grammatical despite vanishingly low probabilities of linear strings (cf. Pereira, 2000; Berwick, 2018)^1^.

In contrast, Lau et al. (2016) recently claimed that the grammar gradiently determines grammatical sentences of the language through probabilities of linear strings without hierarchical structures. On this externalist view, syntactic competence should be reduced to surface linear strings externally attested in large corpora, where grammaticality and acceptability are isomorphic. Specifically, computational models proposed in Natural Language Processing (NLP), such as Markov Models and Hidden Markov Models (HMMs) were trained on large corpora and evaluated against human acceptability judgments via various acceptability measures, demonstrating that probabilities of linear strings can accurately explain human acceptability judgments without hierarchical structures. In response, Sprouse et al. (2018) investigated several computational models evaluated by Lau et al. (2016) with linguistically motivated corpora and measures, and revealed that there are cost-benefit tradeoffs, where computational models accurately explained human acceptability judgments only at the expense of the categorical grammaticality distinction. That is, whether syntactic competence should be characterized by hierarchical structures or reduced to linear strings has been actively debated in the cognitive science literature.

Halle (1973) generalized the internalist view to morphology, and proposed that the grammar (i.e., word formation rules) categorically generates *grammatical* (“potential”) words of the language, whereas humans gradiently judge *acceptable* (“actual”) words of the language, as follows (cf. Aronoff, 1976)^2^:

“In other words, I am proposing that the list of morphemes together with the rules of word formation define the set of *potential* words of the language. It is the filter and the information that is contained therein which turn this larger set into the smaller subset of *actual* words. This set of actually occurring words will be called the *dictionary of the language*.” (Halle, 1973, p. 6; emphasis original)

Embick (2012) corroborated this internalist view of morphology, and suggested that potential words such as *confusal* have the same grammaticality status as the famous *Colorless green ideas sleep furiously* sentence, in that those words are grammatical despite never being attested in large corpora.

However, Bauer (2014) criticized the distinction between grammaticality and acceptability in morphology, and alternatively defended the externalist view of morphology with methodological emphasis on large corpora (cf. Bauer et al., 2013). Indeed, words have been traditionally treated as linear strings of morphemes without any hierarchical structures, as in finite-state models of morphology (Kaplan and Kay, 1994; Beesley and Karttunen, 2003). Moreover, there has been an implicit assumption that words are stored in the mental lexicon without any morpheme units, as in dual-route models of morphology (Pinker and Ullman, 2002) and “amorphous” models of morphology (Baayen et al., 2011).

Nevertheless, there are abundant reasons to believe that morphological competence cannot be reduced to linear strings of morphemes, with apparent differences between syntax and morphology attributed to linguistic performance (cf. Halle, 1973; Bauer, 2014): (i) recursion (e.g., *anti-missile missile*; Bar-Hillel and Shamir, 1960), (ii) center-embedding (e.g., *undeundestabilizablizeable*; Carden, 1983), (iii) long-distance dependency (e.g., *enjoyable*; Sproat, 1992), among other things. Importantly, these morphologically complex words involve nested morphological structures with both prefixes and suffixes and formally require hierarchical structures beyond linear strings (Bar-Hillel and Shamir, 1960; Langendoen, 1981; Carden, 1983). Thus, the nature of morphological competence remains to be empirically investigated.

In this paper, in order to investigate whether morphological competence should be characterized by hierarchical structures or reduced to linear strings, we conduct a crowdsourced acceptability judgment experiment on morphologically complex words and evaluate five computational models of morphological competence against human acceptability judgments. Our morphologically complex words are (i) unattested with zero surface frequencies (i.e., *potential* but not necessarily *actual* words), which increases the possibility that those words have never been encountered by participants and are thus computed from component morphemes, not retrieved from the mental lexicon (cf. Hay, 2003), and (ii) trimorphemic with linear (e.g., *digit-al-ly*) and nested (e.g., *un-predict-able*) morphological structures, the latter of which can only be modeled with hierarchical structures (cf. Libben, 2003, 2006). The computational models investigated in this paper are 1. Character Markov Models (Character) with character linear strings, 2. Syllable Markov Models (Syllable) with syllable linear strings, 3. Morpheme Markov Models (Morpheme) with morpheme linear strings, 4. Hidden Markov Models (HMM) with part-of-speech (POS) linear strings, and 5. Probabilistic Context-Free Grammars (PCFG) with hierarchical structures^3^. Moreover, those computational models are evaluated against human acceptability judgments through the acceptability measure called *syntactic log-odds ratio* (SLOR; Pauls and Klein, 2012) and the evaluation metrics including effect and deviance accuracies, as well as an evaluation metric called *residual accuracy* proposed here to quantify the division of labor among computational models.

This paper is organized as follows. Section 2 describes the crowdsourced acceptability judgment experiment, computational models of morphological competence, and evaluation metrics to statistically compare acceptability judgments and computational models. Section 3 presents descriptive statistics of the acceptability judgment experiment and accuracies of the computational models on several evaluation metrics. Section 4 summarizes and interprets the results in the broader theoretical context. Section 5 concludes this paper.

## 2. Methods

### 2.1. Participants

The participants were 180 native English speakers crowdsourced on Amazon Mechanical Turk (AMT). They provided electronic informed consent and were paid $2/h for their participation. We excluded 14 participants whose native language was not reported to be English (*n* = 5) or whose birthplace was not reported to be the USA (*n* = 9), resulting in 166 participants included in the statistical analyses.

### 2.2. Stimuli

The stimuli were created based on the CELEX lexical database (Baayen et al., 1995). The specific stimuli creation procedure consisted of several steps. First, every word was extracted from the English morphology lemma corpus (eml.cd) available from the CELEX, hence 52,447 words. Second, the words with stem allomorphy (“StrucAllo”), orthographic substitution (“StrucSubst”), or semantic opacity (“StrucOpac”) were excluded, hence 36,800 words. Third, morphological structures (“StrucLab”) were transformed from the CELEX format (e.g., ((teach)[V], (er)[N|V.])[N]) to the Penn Treebank format (e.g., (N (V teach) er)). Fourth, the remaining words were categorized into three types (“MorphStatus”): monomorphemic words (M; *n* = 7,401), zero conversion words (Z; *n* = 7,375), and morphologically complex words (C; *n* = 9,342), which were further subcategorized into bimorphemic words (*n* = 7,383), trimorphemic linear words (*n* = 1,668), and trimorphemic nested words (*n* = 291). The three subcategories of morphologically complex words were defined as [_X_ [_Y_ $\sqrt{\text{Root}}$] Suffix] or [_X_ Prefix [_Y_ $\sqrt{\text{Root}}$]] (bimorphemic), [_X_ [_Y_ [_Z_ $\sqrt{\text{Root}}$] Suffix] Suffix] (trimorphemic linear), and [_X_ Prefix [_Y_ [_Z_ $\sqrt{\text{Root}}$] Suffix]] (trimorphemic nested), where prefixes are attached higher than suffixes. Fifth, trimorphemic linear and nested morphological structures were extracted from trimorphemic linear and nested words, respectively. Specifically, for each outer suffix in trimorphemic linear words (*n* = 48), the possible local combinations with inner suffixes were computed, among which the suffix-suffix combination with the highest type frequency was accepted as trimorphemic linear morphological structure if (i) type frequency ≥5 and (ii) the outer suffix is productive (Plag and Baayen, 2009). In the same vein, for each outer prefix in trimorphemic nested words (*n* = 58), the possible non-local combinations with inner suffixes were computed, among which the prefix-suffix combination with the highest type frequency was accepted as trimorphemic nested morphological structure if (i) type frequency ≥ 2 and (ii) the outer prefix is productive (Zirkel, 2010)^4^. This procedure resulted in 10 linear morphological structures and eight nested morphological structures, as summarized below (N = noun, V = verb, A = adjective, B = adverb):

- Linear morphological structures
  1. [_A_ [_N_ [_V_ $\sqrt{\text{ROOT}}$] ion] al]
  2. [_N_ [_A_ [_V_ $\sqrt{\text{ROOT}}$] able] ity]
  3. [_N_ [_N_ [_V_ $\sqrt{\text{ROOT}}$] or] ship]
  4. [_N_ [_V_ [_A_ $\sqrt{\text{ROOT}}$] ize] er]
  5. [_V_ [_A_ [_N_ $\sqrt{\text{ROOT}}$] al] ize]
  6. [_B_ [_A_ [_N_ $\sqrt{\text{ROOT}}$] ic] ally]
  7. [_B_ [_A_ [_N_ $\sqrt{\text{ROOT}}$] al] ly]
  8. [_N_ [_A_ [_N_ $\sqrt{\text{ROOT}}$] y] ness]
  9. [_N_ [_N_ [_V_ $\sqrt{\text{ROOT}}$] ion] ist]
  10. [_N_ [_A_ [_N_ $\sqrt{\text{ROOT}}$] al] ism]

- Nested morphological structures
  1. [_N_ pre [_N_ [_V_ $\sqrt{\text{ROOT}}$] ion]]
  2. [_A_ sub [_A_ [_N_ $\sqrt{\text{ROOT}}$] al]]
  3. [_A_ super [_A_ [_N_ $\sqrt{\text{ROOT}}$] al]]
  4. [_A_ inter [_A_ [_N_ $\sqrt{\text{ROOT}}$] al]]
  5. [_A_ over [_A_ [_N_ $\sqrt{\text{ROOT}}$] ous]]
  6. [_N_ non [_N_ [_V_ $\sqrt{\text{ROOT}}$] ion]]
  7. [_V_ de [_V_ [_A_ $\sqrt{\text{ROOT}}$] ize]]
  8. [_A_ un [_A_ [_V_ $\sqrt{\text{ROOT}}$] able]]

Finally, novel morphologically complex words were created based on the linear and nested morphological structures generated above. Specifically, for each linear morphological structure, the possible stems were extracted from the subcategory of bimorphemic words whose token frequency is ≥20 and whose inner suffix and syntactic category match with the linear morphological structure. For example, for the linear morphological structure [_A_ [_N_ [_V_ $\sqrt{\text{Root}}$] ion] al], the bimorphemic word *computation* with the structure [_N_ [_V_ $\sqrt{\text{Compute}}$] ion] is the possible stem. Then, one stem was randomly selected from the possible stems and inserted into the linear morphological structure with orthographic adjustments performed (if necessary), and the resultant word was accepted as a novel morphologically complex linear word if unattested in (i) the CELEX lexical database and (ii) the list of socially inappropriate words. Similarly, for each nested morphological structure, the possible stems were extracted from the subcategory of bimorphemic words whose token frequency is ≥ 20 and whose inner suffix and syntactic category match with the nested morphological structure. Then, one stem was randomly selected from the possible stems and inserted into the nested morphological structure with orthographic adjustments performed (if necessary), and the resultant word was accepted as a novel morphologically complex nested word if unattested in (i) the CELEX lexical database and (ii) the list of socially inappropriate words. Importantly, syntactic (i.e., syntactic categories), morphological (i.e., affix combinations), and phonological (i.e., orthographic adjustments) selectional restrictions were explicitly considered, while semantic selectional restrictions were not controlled because those novel morphologically complex words are intended as potential but not actual words, such as *confusal* (Halle, 1973; Embick, 2012)^5^. This final step was repeated until 300 linear and 300 nested trimorphemic words were created, while alternating between linear and nested morphological structures, hence 600 words in total. No roots were repeated in order to avoid potential priming effects across two morphological structures, and those algorithmically generated words were also double-checked by three native English speakers^6^. The stimuli are summarized in Table 1.

**Table 1:** Novel morphologically complex words unattested with zero surface frequencies and trimorphemic with linear and nested morphological structures: 300 linear words (with two inner and outer suffixes) and 300 nested words (with inner suffixes and outer prefixes), hence 600 words in total.

** 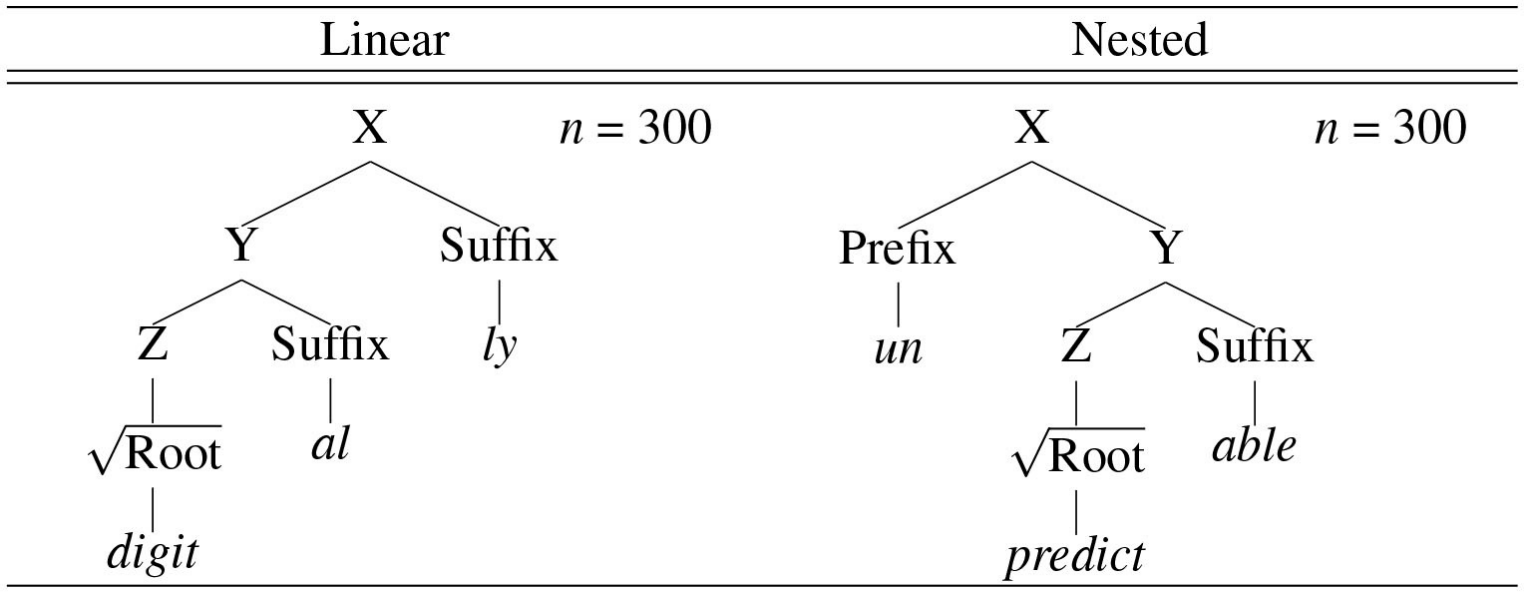 **

### 2.3. Procedure

The 600 novel morphologically complex words were distributed into six different lists of 100 unique words (50 linear and 50 nested words). Each list was randomized and the corresponding reversed list was created, resulting in 12 different lists. Each participant (*n* = 180) was randomly assigned to one of the 12 lists, so that each list was completed by 15 different participants with fixed order. Consequently, there are 30 trials for each word (15 trials from the originally randomized list and 15 trials from the reversed list), hence 18,000 trials (600 words * 30 trials) in total. We excluded 14 participants (*n* = 14 * 100 = 1,400) and incomplete trials (*n* = 61), resulting in 16,539 trials included in the statistical analyses.

The experiment was an acceptability judgment paradigm administered on Amazon Mechanical Turk (AMT) and implemented in HTML, where the participants judged each novel morphologically complex word on the Likert scale from 1 (“very bad”) to 7 (“very good”). In order to ensure that the same participants do not complete the same experiment more than once, the experiment was assigned a unique color code and the AMT workers were asked not to complete the experiments with the same color code more than once per day, given that the entire experiment will be completed within 1 day. Before the experiment, demographic information was collected including gender, age, native language, and birthplace. The instructions are shown below:

“In this experiment, you will read English words, and determine whether you think they are *possible* English words. We are not concerned with whether these words are *actual* English words already listed in a dictionary. Instead, we are interested in whether these words could be used by a native speaker of English. You will rate the word on a scale from 1 (very bad) to 7 (very good). Here are two examples: one that is very bad and one that is very good.”

Importantly, since several pilot experiments suggested that the participants tend to judge novel morphologically complex words based on whether they have ever seen those words before, we explicitly emphasized the contrast between *possible* and *actual* words (Halle, 1973), which encouraged the participants to process the words even if they have never encountered those words before. Then, “very good” (i.e., *teacher*) and “very bad” (i.e., *readize*) bimorphemic examples were presented to familiarize the participants with the Likert scale. Finally, after the additional instruction “There are 100 words for you to rate. You must rate all of them in order to be paid for the experiment,” the experiment started where 100 words were presented with their own Likert scales on the same HTML page. The experiment was piloted with *turktools* (Erlewine and Kotek, 2016) in Python and double-checked by three native English speakers. The experiment lasted for about 10 min^7^.

### 2.4. Computational Models

Five computational models were implemented with Natural Language Tool Kit package (Bird et al., 2009) in Python: Character Markov Model with character linear strings, Syllable Markov Model with syllable linear strings, Morpheme Markov Model with morpheme linear strings, Hidden Markov Model (HMM) with part-of-speech (POS) linear strings, and Probabilistic Context-Free Grammar (PCFG) with hierarchical structures. Those models were trained on the entire CELEX lexical database (*n* = 52,477) via Maximum Likelihood Estimation with token weighting and Lidstone smoothing at α = 0.1, and evaluated against human acceptability judgments of novel morphologically complex words (*n* = 600). The architectures of Markov Model, HMM, and PCFG are summarized below.

#### 2.4.1. Markov Model

Markov Models (also called *n*-gram models) are defined by *n*-order Markov processes that compute transition probabilities of linguistic units (e.g., characters, syllables, morphemes) at position *i* given *i–n* context (e.g., *P*(*x*_*i*_|*x*_*i*−*n*_, *x*_*i*−1_)). Since the length of morphologically complex words is inherently limited relative to syntactically complex sentences, Markov Models were defined with *n* = 1 (i.e., bigram models), which compute transition probabilities of linguistic units at position *i* given the immediately preceding unit (e.g., *P*(*x*_*i*_|*x*_*i*−1_))^8^. For training, Markov Models were unsupervisedly trained on character strings (Character Markov Model), syllable strings (Syllable Markov Model), and morpheme strings (Morpheme Markov Model), respectively, where character and morpheme strings were available from the CELEX lexical database, while syllable strings were generated with the syllabify module implemented in Python by Kyle Gorman through ARPABET transcriptions assigned by LOGIOS Lexicon Tool in the Carnegie Mellon University Pronouncing Dictionary. For testing, those trained Markov Models then computed probabilities of morphologically complex words as products of their component transition probabilities. Markov Models are sequential models, which should accurately predict local dependencies of linear words (e.g., *digitally*), but not non-local dependencies of nested words (e.g., *unpredictable*) because component local dependencies (e.g., *unpredict) are unattested in the training data.

#### 2.4.2. Hidden Markov Model (HMM)

HMMs generalize Markov Models with *n*-order Markov processes defined over “hidden” linear strings. HMMs compute transition probabilities of part-of-speech (POS) tags at position *i* given *i–n* context (e.g., *P*(*t*_*i*_|*t*_*i*−*n*_, *t*_*i*−1_)), and emission probabilities of morphemes at position *i* given POS tags at the same position *i* (e.g., *P*(*m*_*i*_|*t*_*i*_)). Like Markov Models, HMMs were also defined with *n* = 1, which compute transition probabilities of POS tags at position *i* given the immediately preceding POS tag (e.g., *P*(*t*_*i*_|*t*_*i*−1_)). For training, HMMs were supervisedly trained on tagged morpheme strings generated from morphological structures available from the CELEX lexical database (e.g., [(*accident*, N), (*al*, A), (*ly*, B)]). For testing, those trained HMMs then computed probabilities of morphologically complex words as products of component transition and emission probabilities via the forward algorithm which computes the sum of path probabilities of structurally ambiguous words (Rabinar, 1989)^9^. HMMs are also sequential models, which should accurately predict local dependencies of linear words (e.g., N-A-B for *digitally*), but only approximate non-local dependencies of nested words (e.g., *unpredictable*) if component local dependencies (e.g., A-V for *unpredict) are attested in the training data.

#### 2.4.3. Probabilistic Context-Free Grammar (PCFG)

PCFGs generalize Context-Free Grammars (CFGs) with probability distributions defined over hierarchical structures. PCFGs compute non-terminal probabilities of right-hand sides given left-hand sides of non-terminal production rules (e.g., *P*(*rhs*|*lhs*)), and terminal probabilities of right-hand side terminals given left-hand side non-terminals of terminal production rules (e.g., *P*(*m*_*i*_|*t*_*i*_)), equivalent to HMM emission probabilities. Non-terminal production rules are head-lexicalized, which model syntactic selectional restrictions of derivational affixes (e.g., N → A *ness*). For training, PCFGs were supervisedly trained on morphological structures available from the CELEX lexical database (e.g., [_B_ [_A_ [_N_ *accident*] *al*] *ly*]). For testing, those trained PCFGs then computed probabilities of morphologically complex words as products of component non-terminal and terminal probabilities via the Earley parser which computes the sum of tree probabilities of structurally ambiguous words (Earley, 1970; Stolcke, 1995)^10^. PCFGs are hierarchical models, which should accurately predict not only local dependencies of linear words (e.g., [[*digit-al*]*-ly*]), but also non-local dependencies of nested words (e.g., [*un-*[*predict-able*]]).

### 2.5. Statistical Analyses

Mixed-effects regression models were implemented with the lme4 package (Bates et al., 2015) in R. The baseline regression model was first fitted with individual acceptability judgments as the dependent variable (where the acceptability judgments were z-score transformed to eliminate scale biases; Sprouse et al., 2018) and by-subject, by-word, and by-order random intercepts as random effects. Control variables, such as word length and morpheme frequency will be explained by the acceptability measure, thus not included in the baseline regression model. Then, for each computational model, the target regression model was fitted, where the acceptability measure was included as the fixed effect and random effects were held constant. Mixed-effects regression models were fitted via Maximum Likelihood Estimation with nlminb optimizer in optimx package and the maximum number of iterations R permits. Given that the baseline and target regression models are minimally different in the acceptability measure, computational models can be evaluated with nested model comparisons via log-likelihood ratio tests based on the χ^2^-distribution with *df* = 1, where *df* is the difference in the number of parameters between two nested models.

### 2.6. Evaluation Metrics

#### 2.6.1. Syntactic Log-Odds Ratio (SLOR)

The acceptability measure called *syntactic log-odds ratio* (SLOR; Pauls and Klein, 2012) is the linking hypothesis to bridge between probability estimates computed by models and acceptability judgments produced by humans (Lau et al., 2016; Sprouse et al., 2018). SLOR is defined as Equation (1):

(1) $$\begin{array}{r} SLOR=\frac{\log p_{w}\left(\zeta \right)-\log p_{m}\left(\zeta \right)}{\left|\zeta \right|} \end{array}$$

where ζ is the morphologically complex word, |ζ| is the word length, *p*_*w*_(ζ) is the word probability computed by models, and *p*_*m*_(ζ) is the morpheme probability defined as *p*_*m*_(ζ)=∏_*m*∈ζ_*p*(*m*). SLOR was employed in this paper, rather than the mere correlation metric between probability and acceptability, in order to (i) control confounding factors, such as word length (i.e., |ζ|) and morpheme frequency [i.e., *p*_*m*_(ζ)] and focus exclusively on morphological structures, and (ii) keep the evaluation procedure maximally comparable to the previous literature (Lau et al., 2016; Sprouse et al., 2018).

#### 2.6.2. Effect Accuracy

Three evaluation metrics can be derived from SLOR based on effect sizes, deviance statistics, and residual errors. The first evaluation metric called *effect accuracy* is defined as Equation (2):

(2) $$\begin{array}{r} EA\left(model\right)=|d_{human}-d_{model}|=|\Delta d| \end{array}$$

where *d*_*human*_ and *d*_*model*_ are Cohen's *d* estimated from human acceptability judgments and model SLOR scores, respectively, where Cohen's *d* is defined as $d=\frac{\mu_{1}-\mu_{2}}{s}$. That is, the effect accuracy measures the absolute difference in effect sizes between human acceptability judgments and model SLOR scores, so that the lower the effect accuracy is, the more accurate the computational model is (i.e., the computational model with the effect size more comparable to the humans' is more accurate).

#### 2.6.3. Deviance Accuracy

The second evaluation metric called *deviance accuracy* is defined as Equation (3):

(3) $$\begin{array}{r} DA\left(model\right)=D_{base}-D_{model}=\Delta D \end{array}$$

where *D*_*base*_ and *D*_*model*_ are deviance statistics extracted from baseline and target regression models with and without model SLOR scores, respectively, where deviance statistics intuitively quantify the global error between human acceptability judgments and model SLOR scores for each computational model. That is, the deviance accuracy measures the decrease in deviance statistic from baseline to target models, so that the higher the deviance accuracy is, the more accurate the computational model is (i.e., the computational model with lower deviance statistic is more accurate).

#### 2.6.4. Residual Accuracy

The third new evaluation metric called *residual accuracy* is proposed here as Equation (4):

(4) $$\begin{array}{r} RA\left(model\right)=\overset{n}{\underset{i=1}{\sum }}|\epsilon_{base}\left(w_{i}\right)|-|\epsilon_{model}\left(w_{i}\right)|=\overset{n}{\underset{i=1}{\sum }}\Delta |\epsilon \left(w_{i}\right)| \end{array}$$

where ϵ_*base*_ and ϵ_*model*_ are residual errors extracted from baseline and target regression models with and without model SLOR scores, respectively, where residual errors intuitively quantify the local error between human acceptability judgments and model SLOR scores for each morphologically complex word. That is, the residual accuracy can measure the division of labor among computational models with respect to linear and nested morphological structures, so that the higher the residual accuracy is, the more accurate the computational model is (i.e., the computational model with lower residual error is more accurate).

## 3. Results

### 3.1. Descriptive Statistics

**Figure 1:**
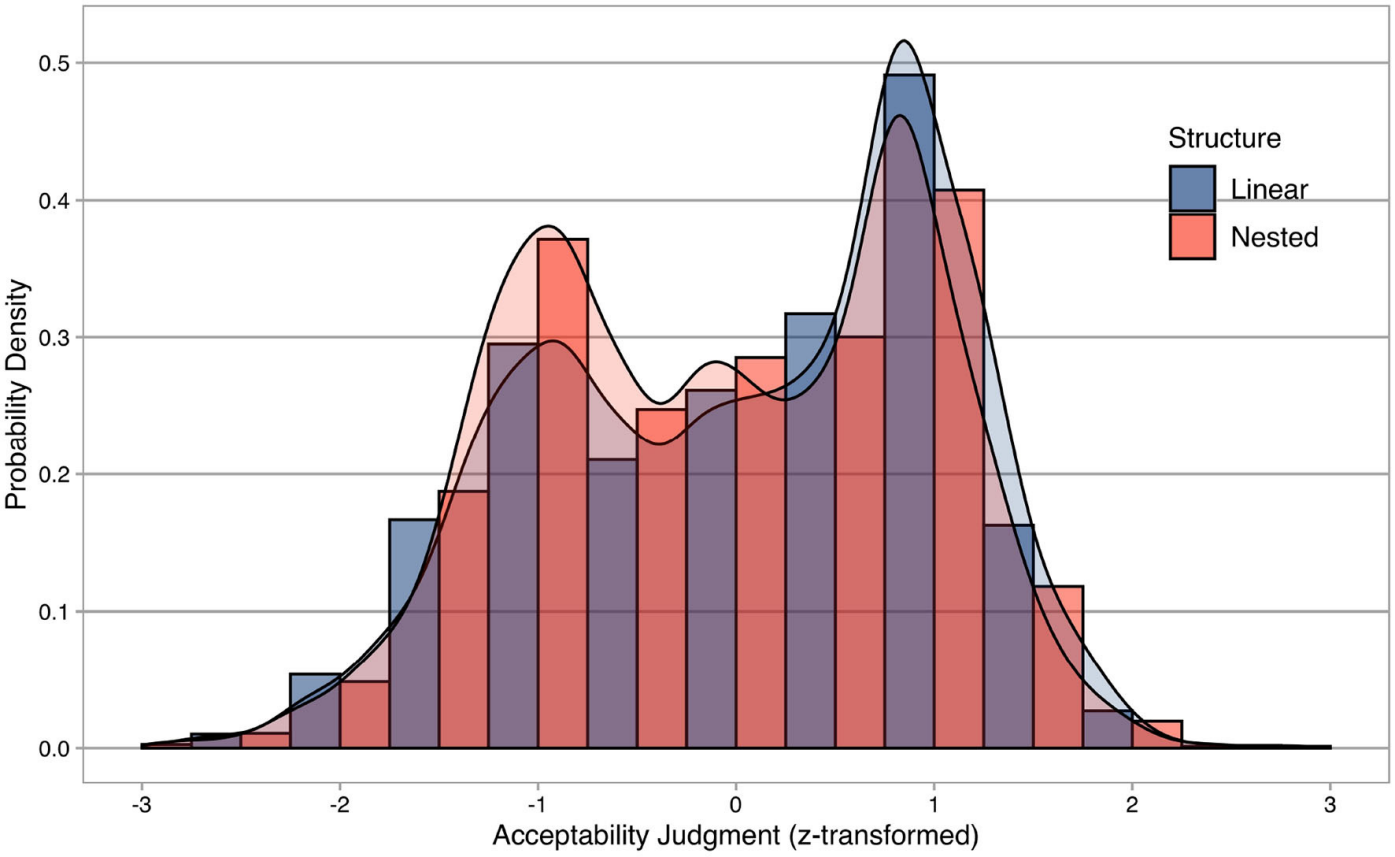
Descriptive statistics of the acceptability judgment experiment. The *x*-axis represents individual acceptability judgments z-score transformed for each participant, while the *y*-axis shows probability densities. Descriptive statistics are separated into linear (blue) and nested (red) structures.

Descriptive statistics of the acceptability judgment experiment are summarized in Figure 1, where the *x*-axis represents individual acceptability judgments z-score transformed for each participant, and the *y*-axis shows probability densities. Descriptive statistics are separated into linear and nested structures.

Importantly, descriptive statistics confirm that the participants were not biased toward only the upper range of the Likert scale, despite the fact that only morphologically complex words (i.e., grammatical words) were tested in this experiment without any morphologically complex nonwords (i.e., ungrammatical words). In addition, the distributions of two morphological structures seem to be bimodal as if both grammatical and ungrammatical words are included in the experiment (cf. Sprouse et al., 2018), suggesting that successful computational models should be balanced and fitted equally well to two morphological structures.

### 3.2. Effect Accuracy

Effect accuracies of computational models are summarized in Table 2, where mean acceptability judgments of linear and nested morphological structures, *t*-values, *p*-values, Cohen's *d*, and effect accuracies (i.e., absolute differences in Cohen's *d* from human acceptability judgments) are presented for each computational model.

**Table 2:** Effect accuracies of computational models.

**Model**	**Linear**	**Nested**	***t***	***p***	***d***	**Δ*d***
Human	4.67	4.39	3.39	<0.001^***^	0.28	—
Character	−6.17	−6.31	0.63	ns	0.05	0.23
Syllable	−1.96	−2.22	0.98	ns	0.08	0.20
Morpheme	2.15	1.47	9.08	<0.001^***^	0.74	0.46
HMM	−0.85	−1.47	11.51	<0.001^***^	0.94	0.66
PCFG	1.35	1.18	2.68	<0.01^**^	0.22	**0.06**

*Mean acceptability judgments of linear and nested morphological structures, t-values, p-values, Cohen's d, and effect accuracies (i.e., absolute differences in Cohen's d from human acceptability judgments) are presented for each computational model*;

** *p < 0.05*,

^**^p < 0.01,

*** *p < 0.001; Bold value represents best performance*.

Independent two-sample *t*-tests indicated that the mean acceptability judgments were significantly different between linear and nested morphological structures for Human (*t* = 3.39, *p* < 0.001***, *d* = 0.28), Morpheme (*t* = 9.08, *p* < 0.001***, *d* = 0.74), HMM (*t* = 11.51, *p* < 0.001***, *d* = 0.94), and PCFG (*t* = 2.68, *p* < 0.01**, *d* = 0.22), where linear morphological structures were judged as more acceptable than nested morphological structures. Among those computational models, PCFG was most accurate with the minimal absolute difference in Cohen's *d* from human acceptability judgments (Δ*d* = 0.06), while Morpheme and HMM were less accurate with the overestimated absolute differences in Cohen's *d* from human acceptability judgments (Δ*d* = 0.46, Δ*d* = 0.66), respectively.

### 3.3. Deviance Accuracy

**Figure 2:**
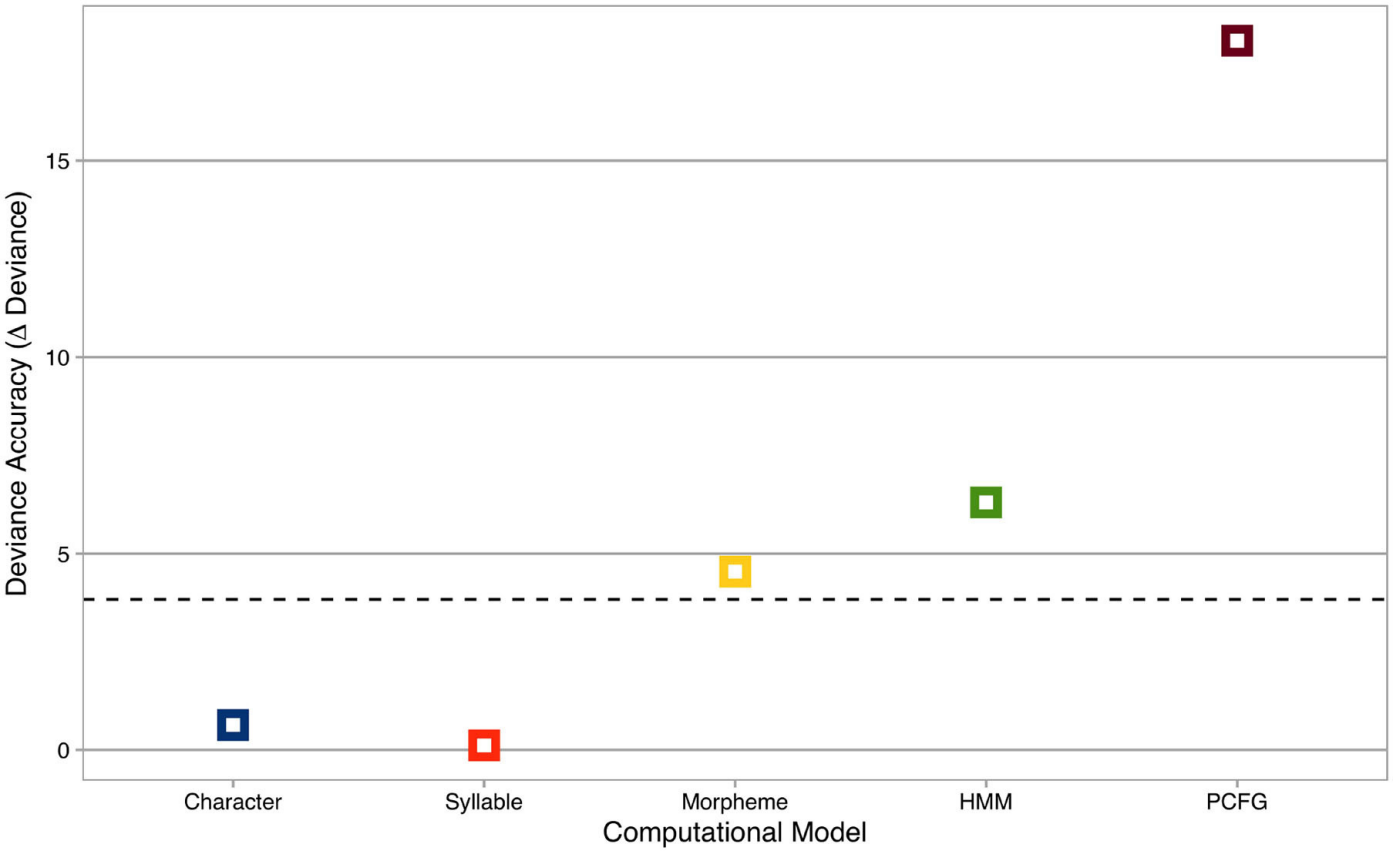
Deviance accuracies of computational models. The *x*-axis represents computational models, while the *y*-axis shows deviance accuracies (i.e., decreases in deviance statistics from the baseline model). Colors indicate computational models: blue = Character Markov Model, orange = Syllable Markov Model, yellow = Morpheme Markov Model, green = Hidden Markov Model, brown = Probabilistic Context-Free Grammar. The horizontal dashed line is χ^2^ = 3.84, the critical χ^2^-statistic at *p* = 0.05 with *df* = 1.

Deviance accuracies of computational models are summarized in Figure 2, where the *x*-axis represents computational models, and the *y*-axis shows deviance accuracies (i.e., decreases in deviance statistics from the baseline model). The horizontal dashed line is χ^2^ = 3.84, the critical χ^2^-statistic at *p* = 0.05 with *df* = 1.

Nested model comparisons revealed that the deviance statistics were significantly different between the baseline model and the target models for Morpheme (χ^2^ = 4.55, *p* < 0.05*), HMM (χ^2^ = 6.3, *p* < 0.05*), and PCFG (χ^2^ = 18.04, *p* < 0.001***). Among those computational models, PCFG was most accurate with the maximal decrease in deviance statistics from the baseline model, while Morpheme and HMM were less accurate with smaller decreases in deviance statistics from the baseline model. In addition, nested model comparisons among computational models confirmed that PCFG significantly outperformed Morpheme (χ^2^ = 13.82, *p* < 0.001***) and HMM (χ^2^ = 11.75, *p* < 0.001***), respectively.

### 3.4. Residual Accuracy

**Figure 3:**
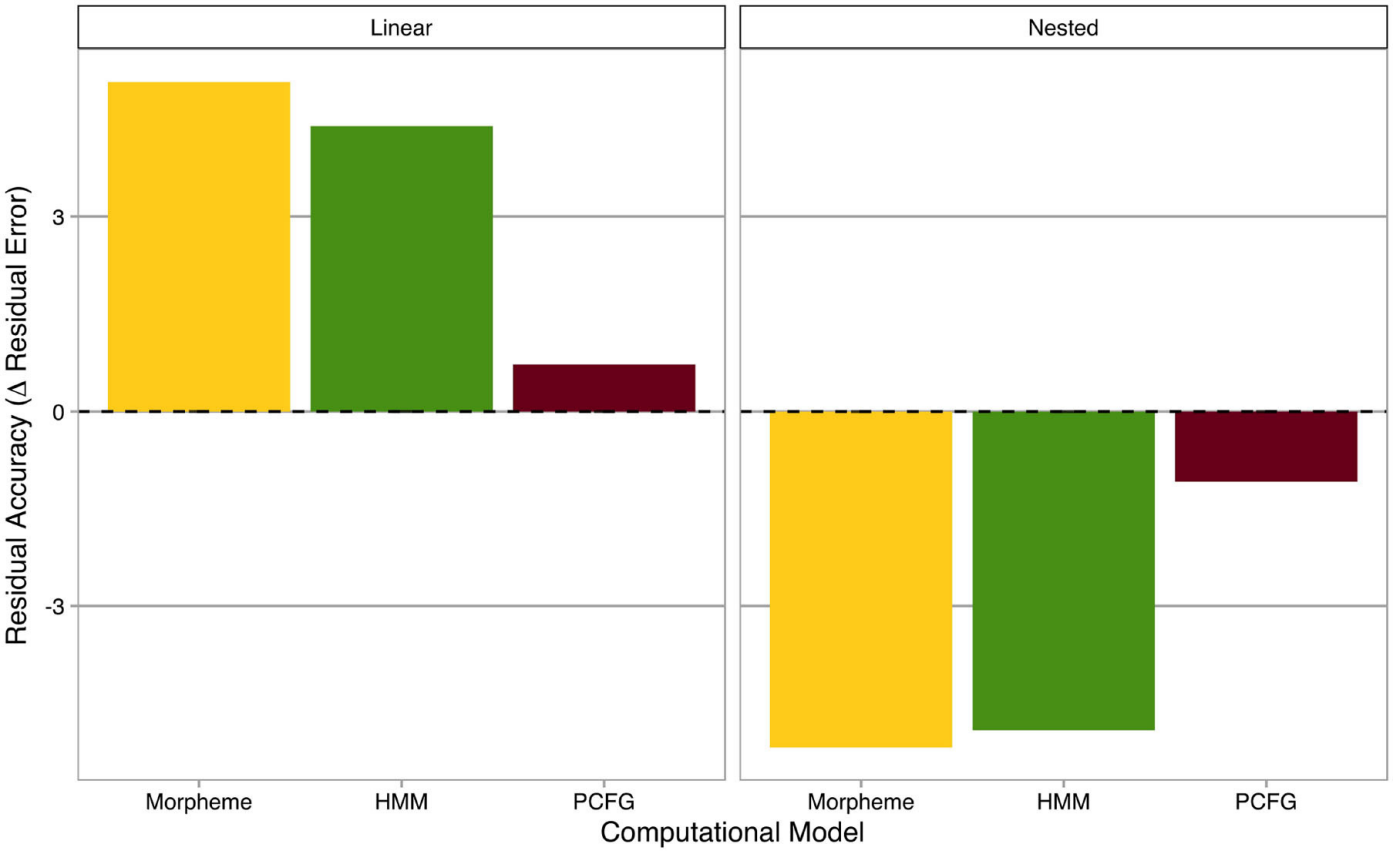
Residual accuracies of computational models. The *x*-axis represents computational models, while the *y*-axis shows residual accuracies (i.e., decreases in absolute residual errors from the baseline model). Residual accuracies are categorized into linear (left) and nested (right) morphological structures. The horizontal dashed line is a “tie” borderline where computational models make the same predictions as the baseline model. Positive and negative residual accuracies mean better and worse predictions relative to the baseline model, respectively.

In order to analyze and interpret the three “morphous” computational models statistically significant on deviance accuracy (i.e., Morpheme Markov Model, HMM, and PCFG), residual accuracies of computational models are summarized in Figure 3, where the *x*-axis represents computational models (without Character and Syllable Markov Models, which were not statistically significant on deviance accuracy), and the *y*-axis shows residual accuracies (i.e., decreases in absolute residual errors from the baseline model). Residual accuracies are categorized into linear and nested morphological structures and averaged across individual derivational affixes. The horizontal dashed line is a “tie” borderline where computational models make the same predictions as the baseline model. Positive and negative residual accuracies mean better and worse predictions relative to the baseline model, respectively.

An interesting mirror image emerged between linear and nested morphological structures. For linear morphological structures, sequential models, such as Morpheme Markov Model and HMM showed higher residual accuracies than the hierarchical model. In contrast, for nested morphological structures, the hierarchical model, namely PCFG, was relatively better than sequential models, although residual accuracies were absolutely negative for all three computational models, potentially suggesting that those computational models were overfitted to linear morphological structures and thus worsened the baseline model.

## 4. Discussion

In summary, we have conducted a crowdsourced acceptability judgment experiment on novel morphologically complex words and then evaluated five computational models of morphological competence against human acceptability judgments via three evaluation metrics. Consequently, both effect and deviance accuracies consistently demonstrated that “morphous” computational models with morpheme units (Morpheme Markov Models, HMM, and PCFG) were more accurate than “amorphous” computational models without morpheme units (Character and Syllable Markov Models). For effect accuracies, “morphous” models correctly predicted the significant differences in effect sizes between linear and nested morphological structures like humans, while “amorphous” models underestimated the differences between those two morphological structures. In the same vein, for deviance accuracies, “morphous” models outperformed “amorphous” models which failed to even reach statistical significance relative to the baseline model. Taken together, these results strongly suggest that morphemes are psychologically real (Marantz, 2013), contrary to “amorphous” models of morphology (Baayen et al., 2011; Ackerman and Malouf, 2013).

More importantly, among those “morphous” models, the hierarchical model, namely PCFG with abstract hierarchical structures, was most accurate on both effect and deviance evaluation metrics as compared to sequential models (Morpheme Markov Model and HMM). For effect accuracies, PCFG most accurately approximated the human effect size between linear and nested morphological structures, whereas sequential models overestimated the effect sizes between those two morphological structures. Similarly, for deviance accuracies, PCFG outperformed sequential models by a large margin. Overall, these results indicate that PCFG is the most “human-like” computational model of morphological competence, contrary to finite-state models of morphology (Kaplan and Kay, 1994; Beesley and Karttunen, 2003)^11^.

Moreover, residual accuracies revealed that there is a division of labor among computational models with respect to linear and nested morphological structures. For instance, sequential models, such as Morpheme Markov Model and HMM accurately explained linear morphological structures at the expense of nested morphological structures. In other words, those sequential models were optimized to linear morphological structures, which naturally follows from their architecture where morphologically complex words are processed incrementally from left to right: linear morphological structures (e.g., *digit-al-ly*) can be predicted from morpheme bigrams of first-second morphemes (e.g., *digit-al*) and second-third morphemes (e.g., *al-ly*) both attested in the training data, while nested morphological structures (e.g., *un-predict-able*) cannot, because morpheme bigrams of first-second morphemes (e.g., *un-predict) never appear in the training data. In contract, the hierarchical model is better balanced and fitted equally well to both linear and nested morphological structures, hence the greater deviance accuracy. Methodologically, this new evaluation metric remains to be adopted in the sentence processing literature to explore the division of labor among computational models for various syntactic constructions (Frank and Bod, 2011; Fossum and Levy, 2012).

Furthermore, remember that novel morphologically complex words were created as *potential* but not necessarily *actual* words (Halle, 1973; Bauer, 2014) with zero surface frequencies in the CELEX lexical database (Baayen et al., 1995) and semantic selectional restrictions not explicitly controlled. To the extent that those morphologically complex words are not stored in the mental lexicon, but rather computed online from component morphemes (cf. Hay, 2003), the fact that humans judged nested morphological structures as acceptable itself constitutes evidence in favor of abstract hierarchical structures.

Finally, we conclude from the results above that there is no fundamental distinction between syntax and morphology, as advocated by the framework of Distributed Morphology (Halle and Marantz, 1993). In formal language theory, given the naive intuition that actual words are stored in the finite lexicon, morphology has been claimed to be finite (in linguistic performance) with respect to weak generative capacity (i.e., string sets generated by the grammar; Langendoen, 1981; Heinz and Idsardi, 2011) and, correspondingly, computationally implemented as finite-state models (Kaplan and Kay, 1994; Beesley and Karttunen, 2003). However, as Carden (1983) correctly pointed out, switching emphasis to strong generative capacity as being only relevant for linguistic theory (i.e., structure sets generated by the grammar; Everaert et al., 2015; Fukui, 2015), morphology turned out to be infinite (in linguistic competence), as exemplified by recursion (e.g., *anti-missile missile*) and center-embedding (e.g., *undeundestabilizablizeable*)^12^. Relatedly, the apparent finite-stateness of morphology gave the impression that morphology is specially sensitive to linear order, but hierarchical structure plays an important role both in syntactic and morphological processing, especially when resolving long-distance dependencies, such as subject-verb agreement in syntax (e.g., *apples on the table are*…vs. *the table are…) and prefix-suffix potentiation in morphology (e.g., *enjoyable*, *joyable). Namely, morphological processing can be regarded as syntactic processing within words.

To recapitulate, going back to the original research question, the results of our psycholinguistic experimentation and computational modeling converged on the conclusion that human morphological competence should be characterized by abstract hierarchical structures, and cannot be reduced to surface linear strings. This conclusion clearly corroborates the internalist view that the grammar generates hierarchical structures (Sprouse et al., 2018), but does not deny probabilities traditionally associated with linear strings (Lau et al., 2016) on the assumption that probability distributions can be defined over hierarchical structures like PCFGs (Yang, 2008). Importantly for the debate between internalist vs. externalist positions, here we advocate the middle position on the spectrum between the extreme internalist (“only grammars, no probabilities”) and extreme externalist (“only probabilities, no grammars”) positions in favor of the eclectic view (Yang, 2004) that grammars (competence) categorically define grammaticality, while probabilities (performance) gradiently affect acceptability.

Nevertheless, there remain several issues with our psycholinguistic experiments and computational models. First, for psycholinguistic experiments, only morphologically complex words (i.e., grammatical words) were tested in this paper, but morphologically complex nonwords (i.e., ungrammatical words) must be developed and tested in order to make the results maximally comparable to the previous literature (Lau et al., 2016; Sprouse et al., 2018). Second, for computational models, Character and Syllable Markov Models were evaluated as instances of “amorphous” models in this paper, but state-of-the-art “amorphous” models, such as Naive Discriminative Learning (Baayen et al., 2011) and Recurrent Neural Network (Kirov and Cotterell, 2018) should be employed and evaluated against human acceptability judgments. Finally, acceptability judgment is known as an offline time-insensitive experimental measure, which only reflects the output of language processing including extra-linguistic factors like working memory and world knowledge (Sprouse, 2007). In order to complement this methodological limitation, novel morphologically complex words developed in this paper must be tested with online time-sensitive experimental measures, such as lexical decision (cf. Oseki et al., 2019).

## 5. Conclusion

In conclusion, we investigated whether human morphological competence should be characterized by abstract hierarchical structures internally generated by the grammar or reduced to surface linear strings externally attested in large corpora. Specifically, we performed a crowdsourced acceptability judgment experiment on morphologically complex words that are (i) unattested with zero surface frequencies and (ii) trimorphemic with linear and nested morphological structures. Then, five computational models of morphological competence were constructed and evaluated against human acceptability judgments via the acceptability measure called *syntactic log-odds ratio*: Character Markov Model (Character), Syllable Markov Model (Syllable), Morpheme Markov Model (Morpheme), Hidden Markov Model (HMM), and Probabilistic Context-Free Grammar (PCFG). Our psycholinguistic experimentation and computational modeling converged on the conclusion that “morphous” computational models with morpheme units outperformed “amorphous” computational models without morpheme units and, importantly, PCFG with hierarchical structures most accurately explained human acceptability judgments via several evaluation metrics, especially for morphologically complex words with nested morphological structures. Those results strongly suggest that PCFG with hierarchical structures is the most “human-like” computational model of morphological competence and, therefore, human morphological competence should be characterized by abstract hierarchical structures internally generated by the grammar.

## Data Availability Statement

The datasets generated for this study are available on request to the corresponding author.

## Ethics Statement

The studies involving human participants were reviewed and approved by New York University's Institutional Review Board (IRB). The patients/participants provided their written informed consent to participate in this study.

## Author Contributions

YO and AM conceived and designed the project, and revised the manuscript together. YO created the stimuli, conducted the experiment, implemented the computational models, performed the statistical analyses, and prepared the manuscript. Both authors contributed to the article and approved the submitted version.

## Conflict of Interest

The authors declare that the research was conducted in the absence of any commercial or financial relationships that could be construed as a potential conflict of interest.

## Footnotes

^1^Due to the ill-posed relationship between grammaticality and acceptability, grammatical sentences may become unacceptable (e.g., garden-path sentences), while ungrammatical sentences can become acceptable (e.g., grammatical illusions).

^2^Halle (1973) proposed that potential words such as *confusal* are assigned the feature [– Lexical Insertion], so that those words can be generated by the grammar, but never inserted into any actual sentences of the language.

^3^Recurrent neural networks (RNNs) were also investigated in the previous literature (Lau et al., 2016; Sprouse et al., 2018), but whether RNNs can implicitly represent hierarchical structures has been intensively debated with mixed results (cf. Linzen et al., 2016; Sennhauser and Berwick, 2018). Thus, as a first approximation, we start with classic but interpretable computational models and leave state-of-the-art but uninterpretable models like RNNs for future research.

^4^The type frequency threshold for nested morphological structures was lower than for linear morphological structures, because the trimorphemic nested words (*n* = 291) were inherently sparse relative to the trimorphemic linear words (*n* = 1,668).

^5^Embick (2012) suggested that those potential words become acceptable if they carve out new “semantic space,” which can be computationally modeled via Functional Representations of Affixes in Compositional Semantic Space (FRACCSS; Marelli and Baroni, 2015), the distributional semantic model which computes meanings of novel morphologically complex words from their component morphemes.

^6^As an anonymous reviewer suggested, the same roots in both morphological structures would help cancel out differences in specific semantic selectional restrictions between roots and inner suffixes across nested and linear morphological structures (e.g., *knowable* vs. **seeable*, as in *unknowable* vs. **seeability*). However, we prioritized not repeating roots within the experiment against controlling this semantic factor across two morphological structures.

^7^While this extended acceptability judgment paradigm might cause the participants to perform meta-linguistic (as opposed to spontaneous) judgments, we decided to adopt this design choice at the expense of spontaneous performance. In addition, the possibility that the same words were re-judged by the same participants multiple times can be safely excluded based on (i) average time per assignment (i.e., 10 min 23 s) and (ii) the incentive of AMT workers (i.e., complete as many assignments as possible).

^8^First-order Markov Models append one word initial symbol < w> as the necessary context to estimate transition probabilities of first morphemes.

^9^We also tested the Viterbi algorithm which computes the max of multiple paths of structurally ambiguous words, but since most probability mass was allocated to the best path, there were no substantial differences between forward and Viterbi algorithms.

^10^In the same vein, we also tested the Viterbi parser which computes the max of multiple trees of structurally ambiguous words, but since most probability mass was allocated to the best tree, there were no substantial differences between Earley and Viterbi parsers.

^11^As an anonymous reviewer correctly pointed out, this conclusion only applies to finite-state *acceptor* models of morphology, but crucially not finite-state *transducer* models of morphology (Kaplan and Kay, 1994; Beesley and Karttunen, 2003), because finite-state transducers can approximate context-free languages of finite length (cf. Langendoen, 1975), such as morphologically complex nested words tested in this paper.

^12^Interestingly, Carden (1983) provided the elaborate context for the example *undeundestabilizablizeable* in order to “assist our feeble performance to reach something closer to the power of the underlying competence” as follows: “At present, gentlemen, we live with an apparently stable balance of terror. But that balance may at any time be de-stabilized by our opponents. As the leaders of a peace-loving state, our objective must be an un-destabilize-able balance. But now, just as we have begun to un-destabilize=able-ize the situation, our opponents have bent all their efforts to de-un=destabilize=able-ize our precarious balance. In our current negotiations, it will not be enough to require an un-destabilize-able balance; we must aim to create an un-de=undestabilizable=ize-able balance.”
